# Monocyte subset recruitment to the peritoneum following abdominal surgical incision in mice

**DOI:** 10.1186/cc9666

**Published:** 2011-03-11

**Authors:** N Bunker, KP O'Dea, JM Handy, M Takata

**Affiliations:** 1 Chelsea & Westminster Hospital, London, UK; 2 Imperial College, London, UK

## Introduction

The current gold standard animal model for sepsis is CLP [1]; however, this model does not allow segregation of the immune responses to infection from those due to surgical incision/trauma. We hypothesised that surgical incision of the peritoneal wall in mice would be a potent stimulus for the recruitment of monocytes, particularlythe inflammatory Gr-1Hi subset [2], to the peritoneal space where they would be capable of mounting a proinflammatory response to subsequent septic challenges.

## Methods

Sterile laparotomy (incision of peritoneum of ~1 cm) was performed on C57B6 mice under isoflurane anaesthesia and closed in two layers. Control groups were skin incision only, or i.p. injection of 20 ng LPS. At least three mice per group were euthanised at intervals up to 48 hours and lavage samples were obtained. For determination of monocyte responses *in situ*, five mice received an i.p. injection of LPS (20 ng) 24 hours post-surgery. Monocyte subset numbers and their expression of the proinflammatory cytokine, TNF, were quantified by flow cytometry.

## Results

In laparotomised mice, migration of Gr-1Hi subset monocytes became evident in lavage fluid at 8 hours, with numbers peaking at 16 hours (7.27 ± 3.25 × 10^5^). Numbers of the Gr-1Lo subset counterpart did not increase until 16 hours but remained high until 48 hours. The peak numbers of both subsets in peritoneal lavage were considerably higher than those observed after i.p. LPS (Gr-1Hi 2.45 ± 1.11 × 10^5 ^and Gr-1Lo 2.69 ± 0.54 × 10^5^). By contrast, skin incision alone did not induce detectable monocyte migration. In response to secondary i.p. LPS challenge, these monocytes recruited by laparotomy responded vigorously, expressing high levels of cell-associated TNF that did not differ significantly between subsets (Gr-1Hi MFI: 146.1; Gr-1Lo MFI: 93.6).

## Conclusions

Monocytes were recruited to the peritoneum in large numbers and for a prolonged period by abdominal surgical incision. The early appearance of the Gr-1Hi followed by Gr-1Lo subset monocytes may represent a delayed kinetic of the latter or the *in situ *maturation of Gr-1Hi to Gr-1Lo monocytes. In view of the numbers recruited and their substantial response to a septic stimulus, monocyte infiltration to the peritoneum could represent a significant risk factor for the development of local and systemic inflammatory conditions following abdominal surgery.
